# Supplementary material for: APP Maturation and Intracellular Localization Are Controlled by a Specific Inhibitor of 37/67 kDa Laminin-1 Receptor in Neuronal Cells
Source: Int J Mol Sci. 2020 Mar 4;21(5):1738. doi: 10.3390/ijms21051738 (PMC7084285; doi:10.3390/ijms21051738)
Supplement: Supplementary file 1 [file ijms-21-01738-s001.pdf]

Supplementary materials

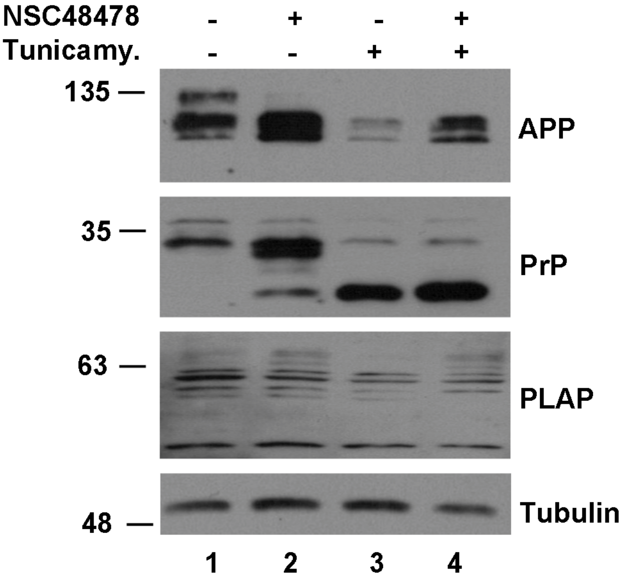

Figure 1. Inhibition of *N*-glycosylation by tunicamycin and specific effects of NSC48478 on APP maturation. GT1 cells, treated or not with NSC48478 for 24h and/or tunicamycin for 16h at 37°C, were processed for western blotting analysis to reveal indicated proteins (APP, PrP, PLAP) in the total (35 μg) cell extract. Anti-tubulin antibody was used to reveal the amount of tubulin in the lysates, as loading control.

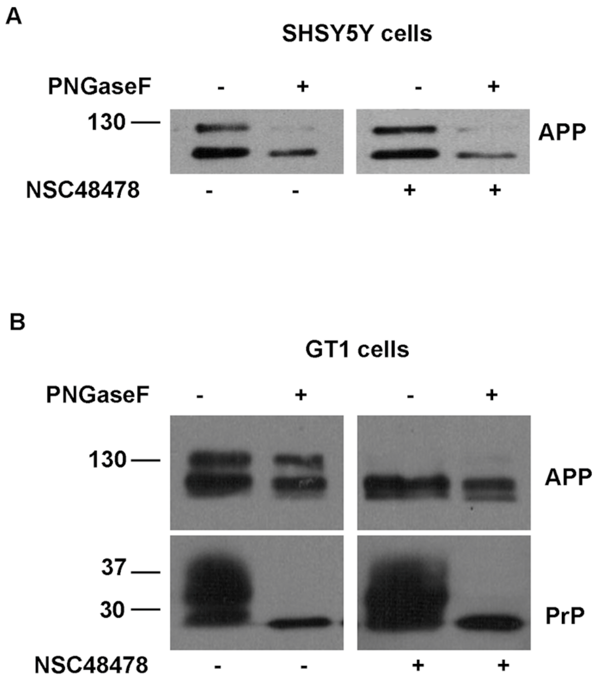

Figure S2. SHSY5Y cells were not affected by NSC48478 and *N*-linked sugar digestion was achieved by PNGaseF in SHSY5Y cells but not in GT1 cells. (A) SHSY5Y cells and **(B)** GT1 cells, treated or not with NSC48478 for 24h, were digested (+) or not (-) with PNGaseF (see details in methods). *N*-glycosylated PrP was carried as control of the procedure.

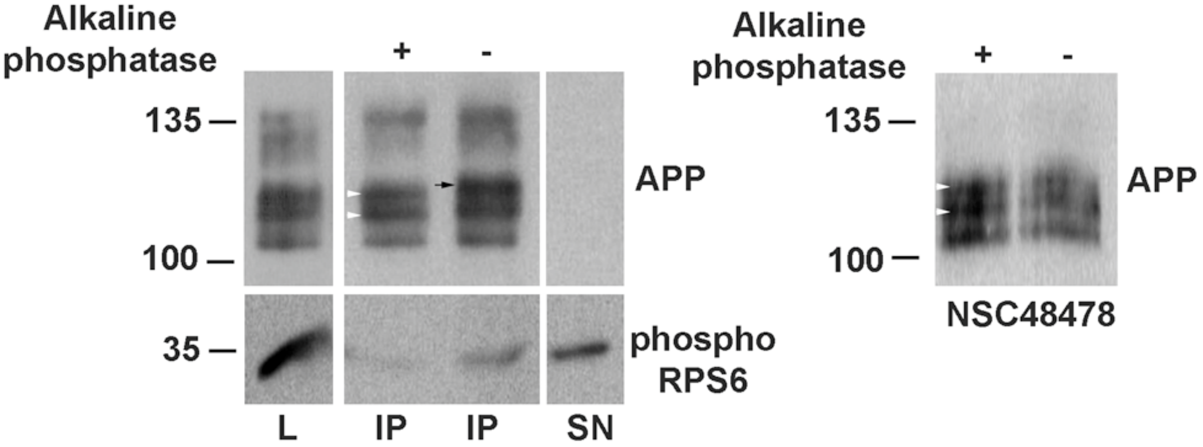

Figure S3. APP is not phosphorylated under NSC48478 treatment. Immunoprecipitated APP from untreated (left panel) or NSC48478-treated GT1 cells (right), was digested with alkaline phosphatase (10U) for 1h at 37°C and run on SDS-PAGE followed by western blotting analysis and hybridization of PVDF membranes with anti-APP antibody. Black arrow indicates phosphorylated APP; white arrowheads point to non-phosphorylated APP. Phospho-ribosomal protein RPS6 was carried as control of the procedure. Note the disappearance of phospho-RPS6 band from the gel after alkaline phosphatase treatment (+). L: cell lysate; IP: immunoprecipitate; SN: supernatant.
